# Identification of potential molecular markers for detection of lengthy chilled storage of *Prunus persica* L. fruit

**DOI:** 10.1016/j.heliyon.2024.e40992

**Published:** 2024-12-05

**Authors:** Giulia Franzoni, Antonella Muto, Leonardo Bruno, Maria Letizia Madeo, Tiziana Maria Sirangelo, Adriana Ada Ceverista Chiappetta, Maria Beatrice Bitonti, Carsten T. Müller, Antonio Ferrante, Hilary J. Rogers, Natasha Damiana Spadafora

**Affiliations:** a Department of Agricultural and Environmental Sciences, University of Milan, Milan, Italy; b Department of Biology, Ecology and Earth Sciences, University of Calabria, Cosenza, Italy; c ENEA-Italian National Agency for New Technologies, Energy and Sustainable Economic Development-Division Biotechnologies and Agroindustry, 00123 Rome, Italy; d School of Biosciences, Cardiff University, Cardiff, United Kingdom; e Department of Chemical, Pharmaceutical and Agricultural Sciences, University of Ferrara, 44121 Ferrara, Italy

**Keywords:** Cold storage, Gene expression, ELISA test, Molecular markers, *Prunus persica* L

## Abstract

Low temperature is the main strategy to preserve fruit quality post-harvest, in the supply chain. Low temperatures reduce the respiration, ethylene emission, and enzymatic activities associated with senescence. Unfortunately, peaches are sensitive to low temperatures if exposed for long periods, resulting in physiological disorders that can compromise commercial quality. Maximum damage occurs at 5 °C while at 1 °C damage is reduced. Therefore, rapid early detection methods for the distribution chain to monitor length and temperature of fruit storage are needed. The aim of this work was to identify candidate genes to develop an antibody-based marker system in peach fruit to monitor chilled storage. Two cultivars were tested: ‘Sagittaria’, an early ripening peach, and ‘Big Top’ a mid-season ripening nectarine, with delayed softening and resistance to supply-chain conditions. Both cultivars were subjected to 1 or 5 °C chilled storage for different times to simulate typical supply-chain conditions. Identification and expression of potential marker genes was assessed using a previous transcriptomic study following storage at 1 °C. Fifteen candidate genes were selected, however only seven proteins encoded were suitable as protein markers as they lack a transmembrane domain. Real-time qPCR using fruit from the subsequent year to the transcriptome was used to assess expression at both 1 and 5 °C chilled storage of five candidate genes. Four genes and the related proteins were identified that would be suitable for the development of molecular markers: a Pathogenesis-Related Bet v I family protein, a dehydrin, a Glycosyl hydrase family 18 protein and a Late Embryogenesis abundant protein.

## Graphical abstract

**Figure:**
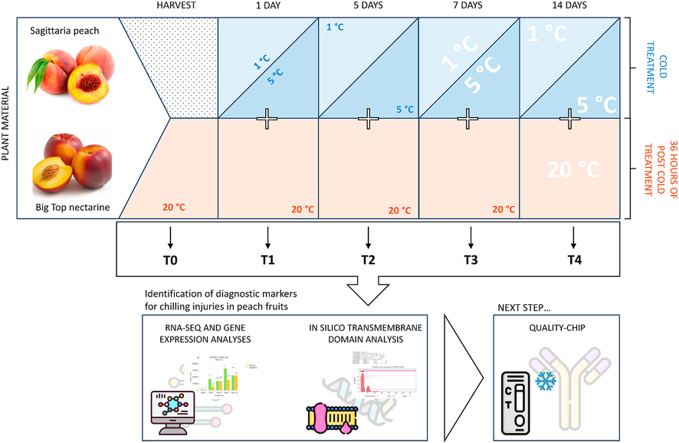

## 1 Introduction

Peach [*Prunus persica* (L.) Batsch] and nectarine [*Prunus persica* (L.) Batsch, var. Nectarina] are important stone fruit crops consumed worldwide. While peaches and nectarines are globally consumed, China, Spain, and Italy accounted for 75 percent of the production in the last decade (2012–2022) [1]. High-quality peaches for fresh market consumption should be firm enough to withstand handling [2], however, their typical soft-flesh and highly perishable nature are limiting factors in shelf life and storage extension [3].

Peaches and nectarines ripen and deteriorate quickly at room temperature, thus low temperature conditions are necessary during transport and to extend fruit commercial life to around 14 d [4]. However, even with sophisticated preservation, transport and storage techniques used to minimize product losses, spoilage during the transport stage is still a major problem [5]. Cold temperatures and prolonged storage can cause alteration of peach fruit quality and ultimately a suite of physiological disorders known as chilling injury (CI) [6]. Chilling injury is a major limiting factor in the life of stored peaches. However, the CI sensitivity has a genetic component: in general, peaches are more often affected by CI than nectarines and most of the mid-season and late-season peach cultivars are more susceptible to CI than early-season cultivars. Chilling injury develops most frequently after storage at temperatures ranging from 2 to 8 °C, during subsequent ripening at room temperature. However, depending on the cultivar, CI can also occur at 0 °C and peach fruit must be stored below 5 °C to delay ripening since temperatures above 5 °C result in rapid tissue softening and quicker ripening [7,8]. Chilled storage also affects aroma profiles and organoleptic quality of the fruit [9]. It would therefore be of benefit to the industry to have markers to monitor chilling periods and temperatures for improving the supply chain management.

Important changes have been detected in the transcriptional profiles of cold stored peaches [[10], [11], [12], [13], [14]] and several studies have been carried out to investigate molecular processes for characterising cold stress tolerance in peaches and nectarines [[15], [16], [17], [18]].

An ideal marker to assess the time x temperature of cold storage would be fast, sensitive, reliable, and low-cost, enabling rapid screening at multiple steps in the supply chain as required. Transcriptional changes can be useful for the identification of clusters of genes associated with quality changes as the accumulation of senescence associated proteins may be linked to quality losses [12]. However, for rapid tests suitable for use in the supply chain, a target protein-based method may be most appropriate, as can be developed for the detection of human patho-genic bacteria in fresh-cut vegetables [19].

Here a comparison was carried out between the early-ripening, melting-flesh, clingstone peach cultivar ‘Sagittaria’, grown in the Calabria, Italy, where fruit typically ripens in May/early June, and the nectarine ‘Big Top’, that ripens in June/July, and is a melting-flesh, semi-free stone fruit. ‘Sagittaria’ is known in the industry to be susceptible to damage from chilled transport, while ‘Big Top’ is a more resilient, widely grown, nectarine cultivar. In a previous study [12] ‘Sagittaria’ lost firmness much earlier than ‘Big Top’ when stored at 1 °C and it was less sweet, more acid, and less juicy after 7 days of storage compared to ‘Big Top’. Based on the data from a transcriptomic study of fruit stored at 1 °C our aim was to identify genes that could be used develop antibody-based marker systems to monitor sub-optimal storage conditions such as long periods of storage and temperature breaches that are associated with development of CI. We also tested whether genes and proteins could be identified that showed sufficiently consistent expression patterns across the two cultivars that they could be used in both, and assessed the patterns at optimal and suboptimal storage temperatures. These findings could be useful for developing a quality management system (QMS) based on the use of objective markers throughout the supply chain. This would be a significant step in ensuring high quality fruit to consumers.

## 2 Materials and methods

### 2.1 Plant material and chilling treatment

‘Sagittaria’ and ‘Big Top’ peaches (*Prunus persica* (L), Batsch] were grown at the “Campo Verde” Agricultural Company in Calabria, Italy [(39°48′58″ N, 16° 12′06″ E, 382 m above sea level (m.a.s.l.),]. Both are yellow melting flesh cultivars; the first one is an early-ripening peach whereas the other is a medium late-ripening nectarine. Peaches were collected at commercial maturity stage in the 2017 and 2018 summer seasons and selected according to appearance [12]. Fruits were sampled before storage (0 d) and stored at 1 °C or 5 °C for 1, 5, 7 and 14 d. Afterwards they were transferred to a growth chamber for acclimatization (22 °C for 36 h). For each time point three biological replicates (each comprising five fruits) were analysed.

### 2.2 Bioinformatic analysis of RNA-seq experiment

Total ribonucleic acid (RNA) extraction, RNA-seq library preparation and the following analyses of differentially expressed genes (DEGs) in ‘Sagittaria’ and ‘Big Top’ samples have been described in detail in a previous paper [12]. Gene expression level values were normalized by the DESEQ2 software and the thresholds used were p value corrected <0.05 and log2FC > 1.5 [14]. Peaches for this study were harvested in 2017. Expression analysis of individual genes used the R package ImpulseDE2 [20]. Detailed statistical analysis of the transcriptome data relative to the expression of each single gene was achieved by ANOVA followed by Tukey's rank test (P < 0.05).

### 2.3 -qPCR analysis

Potential marker genes were identified by bioinformatic analysis performed on the transcriptome profiles of peach stored at 1 °C (Sequence Read Archive database available at NCBI, SRA BioProject PRJNA798864 [12]).

Ribonucleic acid (RNA) was extracted from peaches collected in 2018 from the same location and grower as those used in the transcriptomic analysis, and retro-transcribed into cDNA [18]. Gene expression analyses were performed in triplicates with a StepOne Real-Time PCR instrument (Applied Biosystems, Monza, Italy) by using SYBR Select Master Mix (Applied Biosystem, Monza, Italy). Specific primer pairs for selected genes were designed using Primer3 [[21], [22], [23]] and then Primer-BLAST [24] was used to check primer specificity with “*Prunus persica* (taxid:3760)” as the specified organism (Supplementary Table S1). Polymerase chain reaction (PCR) and gel electrophoresis were used to identify primer pairs that only amplified the intended target and did not dimerize. Primer efficiency was then calculated from a standard curve analysis with −1/slope efficiency were replaced with redesigned primers and then retested.

Amplification reactions were prepared in a final volume of 20 μL by adding 10 μL SYBR Select Master Mix (Applied Biosystems), 2 μL of cDNA template (40 ng/μL), and 1 μL each primer (10 μM). All reactions were run in triplicate in 48-well reaction plates, including negative controls for each target. The qRT-PCR was performed using the following steps: 2 min at 95 C, followed by 40 cycles of 15 s at 95 C and 1 min at 60 °C. Melting curve analysis was also performed at 60 °C for 1 min. PpTEF2 was selected as the endogenous control [25]. Relative quantification of gene expression was calculated using the ΔCT method [26]. Statistical analyses were performed on ΔCt values, first checking for deviations from normality (Kolmogorov-Smirnov test) and tested for homogeneity (Leven Median test) and then analysed by ANOVA and a Tukey's rank test (P < 0.05).

### 2.4 In silico transmembrane domain analysis

After the selection of genes, the putative proteins were analysed in silico to identify those that were not integrated in the membrane using TMHMM-2.0 software (https://services.healthtech.dtu.dk/service.php?DeepTMHMM).

### 2.5 Indirect enzyme-linked immunosorbent assay (ELISA)

Total proteins from fruit mesocarp were extracted from a pool of 5 peaches collected in 2018 from the same location and grower as those used in the transcriptomic analysis, following the protocol reported in the literature [19]. Raw frozen mesocarp (5 g) from the fruits of ‘Sagittaria’ and ‘Big Top’ at commercial harvest (Day 0) and after 5 and 14 days of storage at 5 °C, was ground in pre-chilled mortars with 10 mL of 100 mM K-phosphate buffer (pH 7.8) containing 1 mM EDTA, 25 % (w/v) glycerol, 0.25 % (w/v) Triton X-100, 1 g polyvinylpyrrolidone, 2 mM β-mercaptoethanol, and 1 mM phenylmethylsulfonyl fluoride (86 mM in dimethyl sulfoxide). The homogenate was centrifuged at 15,000×*g* at 4 °C for 30 min, and the supernatant was stored at −80 °C. Total protein content was quantified using the Pierce™ Coomassie Protein Assay Kit (Thermo Scientific), based on Bradford's method. Specific antibodies have been developed against the synthetic peptides (Davids Biotechnologie GmbH) selected by the putative proteins encoded by the target genes. These antibodies have been used to detect the chilling-associated proteins in triplicates using an indirect ELISA method, according to the protocol provided by the company. Absorbances were read with a 405 nm filter using a iMark™ Microplate Absorbance Reader (Bio-Rad).

## 3 Results

### 3.1 Criteria for the initial selection of potential marker genes

**Fig. 1:**
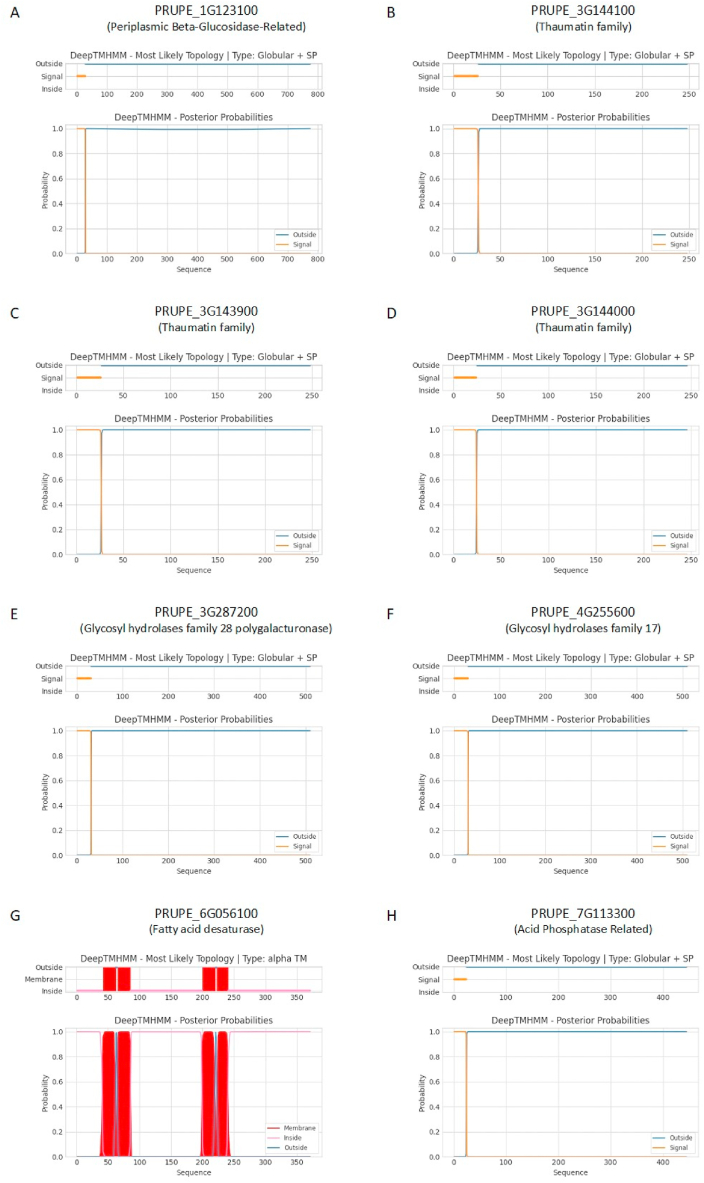
Transmembrane topology prediction of proteins using DeepTMHMM tool. The x-axis represents the length of the protein and the y-axis the probability of a transmembrane domain. (A) Periplasmic beta-glucosidase, (B, C, D) Thaumatin family, (E, F) Glycosyl hydrolases, (G) fatty acid desaturase, (H) Acid phosphatase.

Based on transcriptome sequencing data and bioinformatic analysis in peach cultivar ‘Sagittaria’ and nectarine cultivar ‘Big Top’, 15 genes were identified among the 344 shared DEGs which had the highest increase by day of storage [12] and were included within the 73 DEGs in the “Monotonous Increase” class on the basis of their continuously increasing expression during cold storage at 1 °C over a 14 d period (Supplementary Fig. 1; Supplementary Table S2). A further criterion for the selection of potential marker genes was the assessment of the 15 genes for the inclusion of transmembrane domains in the predicted protein sequences since these might interfere with the production of antibodies [27]. The in-silico analysis revealed that the Periplasmic beta-glucosidase (Prupe.1G123100; Fig. 1 A), three Thaumatin (Prupe.3G144100, Prupe.3G143900, Prupe.3G144000; Fig. 1 B, C, D), two Glycosyl hydrolase (Prupe.3G287200, Prupe.4G255600; Fig. 1E and F), Fatty acid desaturase (Prupe.6G056100; Fig. 1 G), and Acid phosphatase related (Prupe.7G113300; Fig. 1H) predicted proteins did contain a transmembrane domain and therefore were excluded from further analysis (Fig. 1). However, the Glutamine synthetase, Glycosyl hydrolases family 18, Late Embryogenesis Abundant, Dehydrin and three Pathogenesis related genes did not encode proteins with transmembrane domains. Among the three Pathogenesis-related proteins, only the DEG of greatest significance, as identified in the transcriptomic profiling study by Muto et al., 2022 [12], was chosen for further investigation.

### 3.2 Evaluation of potential markers based on transcriptomic dataset

**Fig. 2:**
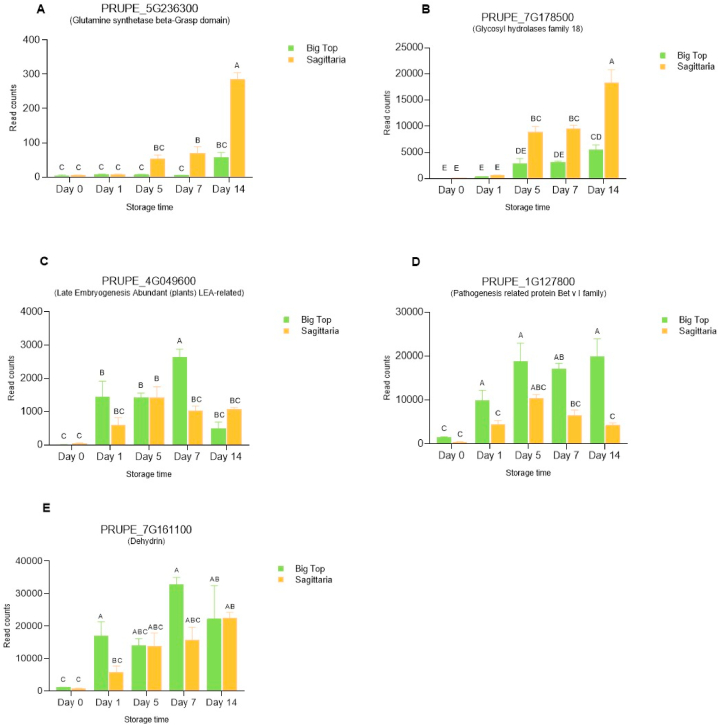
Gene expression changes in Sagittaria peach and Big Top nectarine during cold storage treatment at 1 °C at 0, 1, 5, 7 and 14 d followed by 36 h recovery at ambient temperature (22 °C) in 2017 based on transcriptomic analysis. Differentially expressed genes (DEGs) during the time course are expressed as read counts and obtained by ImpulseDE2. (A) Glutamine synthetase beta-Grasp domain, (B) Glycosyl hydrolases family 18, (C) Late Embryogenesis Abundant (plants) LEA-related, (D) Pathogenesis related protein Bet v I family, (E) Dehydrin. Different letters indicate significant differences among cultivars considering all time points. Statistical analyses were performed using Two-way ANOVA and Tukey's ranked test (P < 0.05). Data are the mean ± SE; n = 3.

A detailed statistical analysis of the transcriptome data for the 5 potential markers genes revealed that the expression of two of the genes: Glutamine synthetase beta-Grasp domain, Glycosyl hydrolases family 18 was significantly higher in ‘Sagittaria’ peach than in ‘Big Top’ nectarine, after day 5 of storage (2.98 and 1.63 log2FC respectively), especially at 14 d of storage where the log2FC were 2.32 and 1.73 respectively (Fig. 2 A, B and Supplementary Table S3).

The expression of Late Embryogenesis Abundant (plants) LEA-related, Pathogenesis related protein Bet v I family, and Dehydrin showed different trends. Late Embryogenesis Abundant (plants) LEA-related gene increased in ‘Big Top’ fruit up to 7 d of storage, with a higher expression at day 7 compared to ‘Sagittaria’ (−1.35 Log2FC), and then decreased at 14 d. In ‘Sagittaria’ fruit the expression level peaked at 5 d and remained constant thereafter (Fig. 2C). The expression of Pathogenesis related protein Bet v I family in ‘Sagittaria’ increased after harvest with a 3.3 Log2FC after 1 d of storage and was fairly constant throughout storage (Fig. 2D–Supplementary Table S2). In ‘Big Top’ expression was significantly higher (2.78 Log2FC) at 1 d onwards compared to harvest (Fig. 2D–Supplementary Table S2). The expression of Dehydrin showed a similar trend in both cultivars characterised by a constant increase during storage. There was no significant difference between the two cultivars, apart from at day 1 where the expression was significantly higher in ‘Big Top’ fruit than in ‘Sagittaria’ with a −1.56 Log2FC (Fig. 2E–Supplementary Table S3).

### 3.3 Selected potential markers during chilled storage at two storage temperatures

**Fig. 3:**
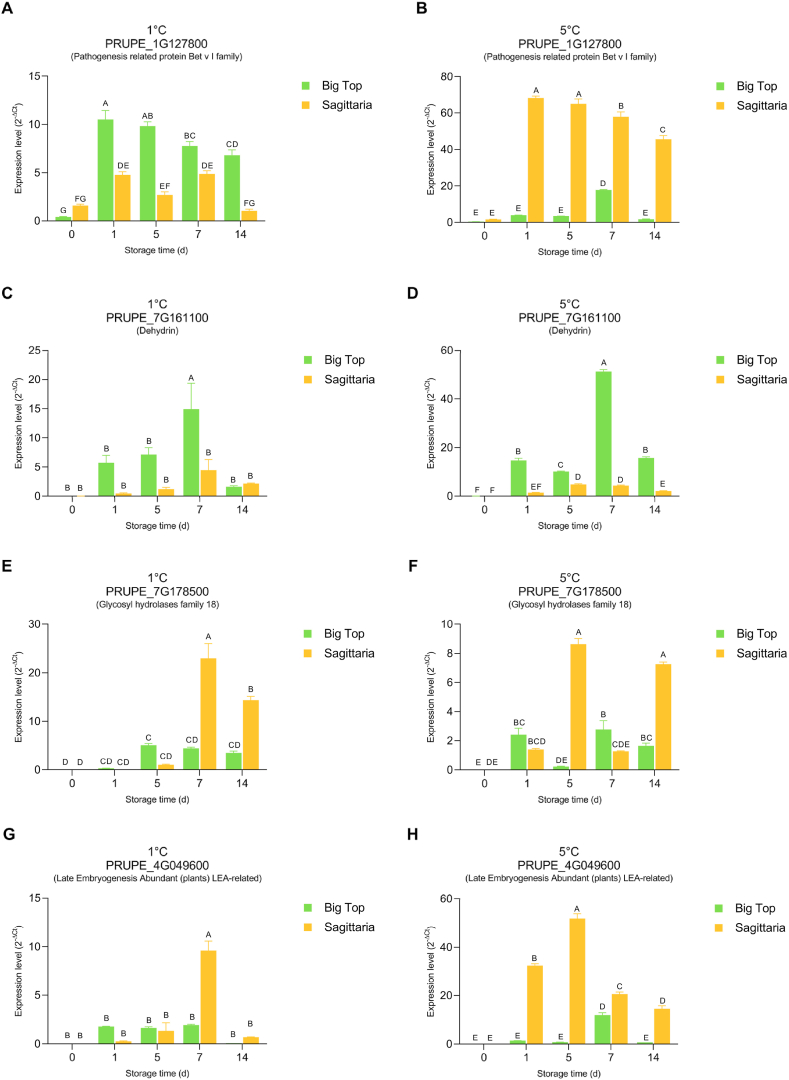
Real-time PCR analysis of selected DEGs in Sagittaria peach and Big Top nectarine during cold storage treatment at 1° (A, C, E, G) and 5 °C (B, D, F, H) at 0, 1, 5, 7, and 14 d followed by 36 h recovery at ambient temperature (22 °C). Relative expression (2^−ΔCt^): (A) and (B) Glycosyl hydrolases family 18; (C) and (D) Late Embryogenesis Abundant (plants) LEA-related; (E) and (F) Pathogenesis related protein BET v I family, (G) and (H) Dehydrin. Different letters indicate significant differences among cultivars considering all time points. Statistical analyses were performed using Two-way ANOVA and Tukey's ranked test (P < 0.05). Data are the mean ± SE; n = 3.

Reverse transcription-quantitative real-time PCR (RT-qPCR) was used to assess expression of the selected genes in ‘Sagittaria’ peach and ‘Big Top’ nectarine at harvest and stored fruit at both 1 and 5 °C for 14 d. Importantly, fruit from the subsequent year were used to verify if the expression pattern of these genes was consistent across seasons (Fig. 3). Compared to the transcriptomic data (Fig. 2), the Pathogenesis related protein Bet v I family (Prupe.1G127800) showed a very similar expression pattern in ‘Big Top’ using RT-qPCR with a dramatic increase between 0 and 1 d of storage at 1 °C (4.67 Log2FC, Fig. 3A and Supplementary Table S4). However, in contrast to the transcriptomic data, the RT-qPCR indicated a significant fall in expression of this gene over the 14 d storage period although expression remained significantly higher compared to 0 d expression (Fig. 3 A). In ‘Sagittaria’ RT-qPCR of the same gene also confirmed the overall pattern with a significant rise in the first day of storage and a 1.58 Log2FC. Thereafter a broadly constant but lower expression than in ‘Big Top’ and a decrease by 14 d which compared to Day 7 showed a −2.2 Log2FC (Fig. 3 A and Supplementary Table S4). The pattern of expression during storage at 5 °C was remarkably different from that at 1 °C (Fig. 3 B). At 5 °C, expression was much higher in ‘Sagittaria’ than ‘Big Top’ (Log2FC of 1.7; Fig. 3B and Supplementary Table S5) as was the increase in expression between 0 and 1 d of storage with a Log2FC of 5.41 and 3.21, respectively. Although there was a significant increase in expression at 7 d of storage in ‘Big Top’ (2.38 Log2FC) on other storage days the expression was not significantly higher than at 0 d (Fig. 3 B).

The pattern of expression of dehydrin (Prupe.7G161100) was also broadly confirmed by the RT-qPCR in the following year of harvest with a strong increase in expression in the first storage day and a fall by 14 d for ‘Big Top’ at 1 °C with Log2FC of 6.92 and −3.2, respectively (Fig. 3C and Supplementary Table S4). In ‘Sagittaria’ the RT-qPCR indicated that there was a rise in expression during storage but the rise in expression was much less clear than seen in the transcriptome data set (Fig. 3C and Supplementary Table S4). In contrast to the Bet v-like gene expression, the expression of the dehydrin gene during 5 °C storage was similar to that seen at 1 °C (Fig. 3 D and Supplementary Table S4).

The RT-qPCR confirmed the expression pattern of the Glycosyl hydrolase family 18 gene (Prupe.7G178500) seen in the transcriptomic data set at 1 °C and late storage time with a rise in expression in ‘Sagittaria’ with 2.05 Log2FC by day 14 compared to ‘Big Top’(Fig. 3 E and Supplementary Table S5). In contrast to storage at 1 °C, storage at 5 °C elicited increases in expression in both ‘Big Top’ and ‘Sagittaria’ earlier in the storage period at 1 d with 12.93 and 6.2 Log2FC, respectively (Fig. 3 F and Supplementary Table S4).

The expression pattern of the LEA protein (Prupe.4G049600) at 1 °C was somewhat different from the RT-qPCR data compared to the transcriptome data. In ‘Big Top’ although there was a slight rise in expression during storage, it was not significant, and in ‘Sagittaria’ the rise in expression was only significant at 7 d instead of 5 d (Fig. 3 G). At 5 °C expression rose in both cultivars but the rise in expression in ‘Sagittaria’ was much earlier at 1 d of storage (11.14 Log2FC), and in ‘Big Top’ the rise in expression was significant after 7 d of storage (3.92 Log2FC) (Fig. 3H and Supplementary Table S4). The expression level of glutamine synthetase β-grasp domain gene (Prupe.5G236300) was below detection in both cultivars and at storage both at 1 °C and 5 °C and therefore excluded from further analysis. This lack of detection may be due to the low expression of this gene seen as very low read counts compared to the other genes in the transcriptome (Fig. 2).

A preliminary test on the accumulation of proteins encoded by the genes studied, as validation of the consistency of the markers associated with the chilling was made using ELISA. Polyclonal antibodies were used for the protein detection and the validation of proteins as putative markers for prolonged chilled storage was performed at 5 °C on Pathogenesis related protein Bet v I family, Dehydrin, Glycosyl hydrolases family 18, and Late Embryogenesis Abundant (plants) LEA-related proteins. The patterns of change showed a high degree of concordance between protein and gene expression for the Bet V protein in both cultivars. For the other genes there was a similar trend, with a consistent early increase from 0 to 5 d, but in the case of the protein the accumulation continued or plateaued after day 5 whereas in some cases the gene expression peaked at 5 or 7 d and then fell by day 14 (Supplementary Fig. 2).

## 4 Discussion

Based on sequence data and bioinformatic analysis of transcriptomic data from *P. persica* [12] the fifteen genes selected were potentially suitable for use as markers of prolonged cold storage. The temperature used in the transcriptomic study was selected to simulate optimal storage transport conditions in the supply chain [8] The 14 d period is typically before CI develops [6], so these markers would not be specifically predictive of CI but could act as markers that indicate prolonged storage and therefore a higher risk of developing CI later in the supply chain.

However, eight of these genes were predicted to have a transmembrane domain. Unfortunately, it can be difficult to detect transmembrane proteins using target-molecular tests [27,28], therefore these genes may not be the best targets for developing an antibody-based marker system. Of the remaining genes, seven showed no evidence of encoding a protein with membrane localization, and verification of the expression patterns for five of these was attempted, to assess expression across seasons. Two different storage temperatures were assessed: optimal at 1 °C and sub-optimal at 5 °C [7,8]. These five genes were annotated as: Glutamine synthetase, beta-Grasp domain (also called glutamine synthetase nodule isozyme; Prupe.5G236300), Late Embryogenesis Abundant (plants) LEA-related (also known as group 3 LEA protein; Prupe.4G049600), Dehydrin (Prupe.7G161100), Glycosyl hydrolases family 18 (Prupe.7G178500) and Pathogenesis related protein Bet v I family (Prupe.1G127800). All five genes were reported as having roles in stress responses, consistent with an increase in their expression during chilled storage of peach fruit. Glutamine synthetase (Prupe.5G236300) is a key enzyme in the process of nitrogen assimilation and remobilization in plants, leading to the formation of glutamine [29]. This enzyme is important for the remobilization of nitrogen during natural leaf senescence [30]. The level of glutamine synthetase transcripts does not vary significantly during the development of the peach fruit but increases strongly during post-harvest ripening accompanied by an increase in glutamate dehydrogenase (GDH) expression [31]. During chilled fruit storage, glutamine synthetase might be part of a pathway involved in the conversion of protein amino acids into non-protein amino acids, such as amino butyric acid (GABA), as well as proline, compounds that act as signals or in the protection of macromolecules since these metabolites increase in fruit in response to cold stress [32,33]. Late Embryogenesis Abundant (LEA) proteins make up a very large and variously differentiated family of proteins with antioxidant properties and can bind metals, stabilize the structure of proteins and membranes while maintaining their hydration. They interact with DNA and RNA and are often induced in response to abiotic stresses. Both Prupe.4G049600 and Prupe.7G161100 belong to this family of proteins. The latter belongs to the dehydrin LEA group, that was identified in a proteomic analysis of mealiness in peach fruit [34] and may have a role in promoting tolerance to low temperatures by binding to membranes and improving membrane stability [34,35] Prupe.4G049600 is most similar to Arabidopsis At5g44310 a member of the LEA-4 protein group [36]. At least one LEA-4 protein, from Medicago sativa [37] was shown to confer cold stress tolerance. Glycosyl hydrolases family 18 (Prupe.7G178500) is an endochitinase. In plants endochitinases are thought to play a role in defence against chitin containing pathogens [38] but may also have other functions in stress responses. In peach fruit this gene has been associated with resistance to UVC stress [39]. Pathogenesis related Bet v I family (Prupe.1G127800) proteins are similar to MLP-like protein 423 of which some are involved in drought tolerance and fruit ripening [40,41].

From a detailed analysis of the transcriptome data (Fig. 2), the most promising candidates were the glutamine synthetase gene (Prupe.5G236300) and the glycosyl hydrolase gene (Prupe.7G178500) both of which are maximally expressed late in the storage period in both peach and nectarine cultivars tested, with a strong up-regulation compared to the freshly harvested fruit. These would therefore be suitable to report on length of storage period. The other three genes considered had more complex patterns of expression that differed amongst the two cultivars. However, it was important to verify these patterns by qPCR using material from a different season.

Unfortunately, it was not possible to assess the expression of the glutamine synthetase gene (Prupe.5G236300) using real-time qPCR as expression was too low, as is seen in the low read counts (Fig. 2A). This gene was therefore eliminated from the potential marker panel. Real-time qPCR was however successful for the remaining four genes.

Expression of the PR-related Bet v family protein (Prupe.1G127800) peaked early with different responses in the two cultivars dependent on temperature of storage. This protein was previously identified as a major peach fruit allergen (PR-10 or Pru p1 [42]) present in the pulp and peel of peach [43]. In previous work, *Pru P1* expression increased at 0 °C during storage but fell between 14 and 21 d of storage when peach fruit were stored at 5 °C [44] and the authors suggested a role for this protein in the stabilization of membranes at low temperatures. Here, expression of this gene rose in the first few days of storage in both cultivars at 1 °C and in both years. In both cultivars expression fell at 5 °C between days 7 and 14, also in line with the published data [44]. However, expression in both cultivars fell later in the storage period when fruit were, stored at 1 °C which is not consistent with previous data [44]. It is possible that the expression pattern at the lower temperature is complex, and that expression rises again after 14 d also in the cultivars studied here or the rise in expression at 1 °C reported previously may be related to the cultivar used (cv. Hongtao) [44]. Further data would be required to clarify this. Another feature of the expression pattern seen here was a much higher expression in ‘Sagittaria’ fruit stored at 5 °C compared to 1 °C. As ‘Sagittaria’ is much more sensitive to chilling [12] the expression pattern of this gene indicates that it might be a useful marker for detecting changes in storage temperature in more CI sensitive cultivars, although verification of its expression pattern in more cultivars and at a protein level would be needed.

The dehydrin gene (Prupe.7G161100) increased in expression in both peach cultivars with cold storage peaking at 5–7 d. Notably expression was much higher in the more resilient ‘Big Top’ at both storage temperatures which is consistent with previous studies associating dehydrin expression and stress tolerance [35]. Expression of one dehydrin gene, *PbDHN5* was in pear fruit conditioned to reduce CI during storage whereas other dehydrin genes were down-regulated suggesting a role in chilling responses in other fruit [45]. Here the dehydrin expression rose quite early during storage and fell off by 14 d of storage at both temperatures tested [45]. It therefore may be less useful as a marker for prolonged cold storage.

The glycosyl hydrolase gene (Prupe.7G178500) was upregulated by the chilled storage, consistent with a role in stress responses previously suggested [39]. In the first year of analysis expression continued to rise in both cultivars up to the end of the 14 d test period suggesting it might be a useful gene for charting the length of storage. Late upregulation was seen in both cultivars and especially in ‘Sagittaria’ at 1 °C in the first season tested although in the second season expression fell off by 14 days. However, at 5 °C expression rose much earlier; this might make it a useful marker for detecting changes in storage temperature profile.

Consistent with a possible role of LEA-4 group proteins in conferring cold tolerance [37], Prupe.4G049600 was strongly and rapidly upregulated by the cold storage in both cultivars in the first year of study. However, in the subsequent year upregulation was only detected much later in ‘Big Top’ at 5 °C at and 1 °C upregulation in ‘Sagittaria’ was only detected after 1 week of storage. In both cultivars, at both temperatures and in both years of study expression declined by 14 d of storage. In ‘Sagittaria’, however, expression was much higher when fruit were stored at 5 °C than 1 °C. So, this gene might be suited for detecting temperature changes during storage rather than reporting on long periods of storage.

The preliminary validation of proteins as putative markers of prolonged chilled storage was performed on Pathogenesis related protein Bet v I family, Dehydrin, Glycosyl hydrolases family 18, and Late Embryogenesis Abundant (plants) LEA-related. Results indicated that gene expression and proteins showed the same trend, and indeed the protein continued to accumulate with longer periods of storage. This suggests promising applications in postharvest for early detection of storage conditions that favour the development of chilling injury.

## 5 Conclusion

In this study four genes have been identified as potential target for developing a molecular-based marker system for charting storage, and which may be useful in quality assessment since CI is most associated with longer periods of storage at temperatures above 1 °C. This work can be considered as a first step developing antibody-based markers and rapid tests for verifying length of chilled storage and cold-chain disruptions, which can be then associated later with the development of CI. Moreover, this work stresses the importance of verifying expression in different cultivars and across multiple years of study and assessing suitability for use in tests. It also suggests that a combination of markers may be most useful to assess both storage time and temperature. To better understand the general applicability across various peach varieties these preliminary results need further validation on additional peach cultivars and with different cold stress tolerance traits.

## CRediT authorship contribution statement

**Giulia Franzoni:** Writing – review & editing, Writing – original draft, Investigation, Formal analysis. **Antonella Muto:** Writing – review & editing, Investigation, Formal analysis. **Leonardo Bruno:** Writing – review & editing, Supervision, Conceptualization. **Maria Letizia Madeo:** Writing – review & editing, Investigation, Formal analysis. **Tiziana Maria Sirangelo:** Writing – review & editing, Investigation, Formal analysis, Data curation. **Adriana Ada Ceverista Chiappetta:** Writing – original draft. **Maria Beatrice Bitonti:** Writing – original draft. **Carsten T. Müller:** Writing – review & editing. **Antonio Ferrante:** Writing – review & editing, Writing – original draft, Supervision, Investigation, Formal analysis, Data curation, Conceptualization. **Hilary J. Rogers:** Writing – review & editing, Writing – original draft, Supervision, Investigation, Formal analysis, Data curation, Conceptualization. **Natasha Damiana Spadafora:** Writing – review & editing, Writing – original draft, Supervision, Project administration, Methodology, Investigation, Funding acquisition, Formal analysis, Data curation, Conceptualization.

## Ethical approval statement

Not applicable.

## Data availability statement

The data and material generated during and/or analysed during the current study are available from the corresponding author upon reasonable request.

## Funding

This research was supported by Fondazione con il Sud, call Brain2South, as part of the FRUITY collaborative project (2015–0245). NDS also benefits from funding of the program PON “Research and Innovation” 2014–2020 (PON R&I), Action IV.6 “Contratti di ricerca su tematiche Green”.

## Declaration of competing interest

The authors declare the following financial interests/personal relationships which may be considered as potential competing interests:Natasha Damiana Spadafora reports financial support was provided by CON IL SUD Foundation. Natasha Damiana Spadafora reports financial support was provided by PON. If there are other authors, they declare that they have no known competing financial interests or personal relationships that could have appeared to influence the work reported in this paper.
