# Supplementary material for: Identification of potential molecular markers for detection of lengthy chilled storage of Prunus persica L. fruit
Source: Heliyon. 2024 Dec 5;10(24):e40992. doi: 10.1016/j.heliyon.2024.e40992 (PMC11667614; doi:10.1016/j.heliyon.2024.e40992)
Supplement: Multimedia component 1 [file mmc1.docx]

Identification of potential molecular markers for detection of lengthy chilled storage in *Prunus persica* L. fruit

Giulia Franzoni^a#^, Antonella Muto^b#^, Leonardo Bruno^b^, Maria Letizia Madeo^b^, Tiziana Maria Sirangelo^c^, Adriana Ada Ceverista Chiappetta^b^, Maria Beatrice Bitonti^b^, Carsten T Müller^d^, Antonio Ferrante^a^, Hilary J Rogers^d^, Natasha Damiana Spadafora^e^*

^a^Department of Agricultural and Environmental Sciences, University of Milan, Milan, Italy

^b^Department of Biology, Ecology and Earth Sciences, University of Calabria, Cosenza, Italy

^c^ENEA-Italian National Agency for New Technologies, Energy and Sustainable Economic Development-Division Biotechnologies and Agroindustry, 00123 Rome, Italy

^d^School of Biosciences, Cardiff University, Cardiff, United Kingdom

^e^Department of Chemical, Pharmaceutical and Agricultural Sciences, University of Ferrara, 44121 Ferrara, Italy

^#^These authors contributed equally to this work

*Corresponding author: Natasha D Spadafora [damiana.spadafora@unife.it](mailto:damiana.spadafora@unife.it)


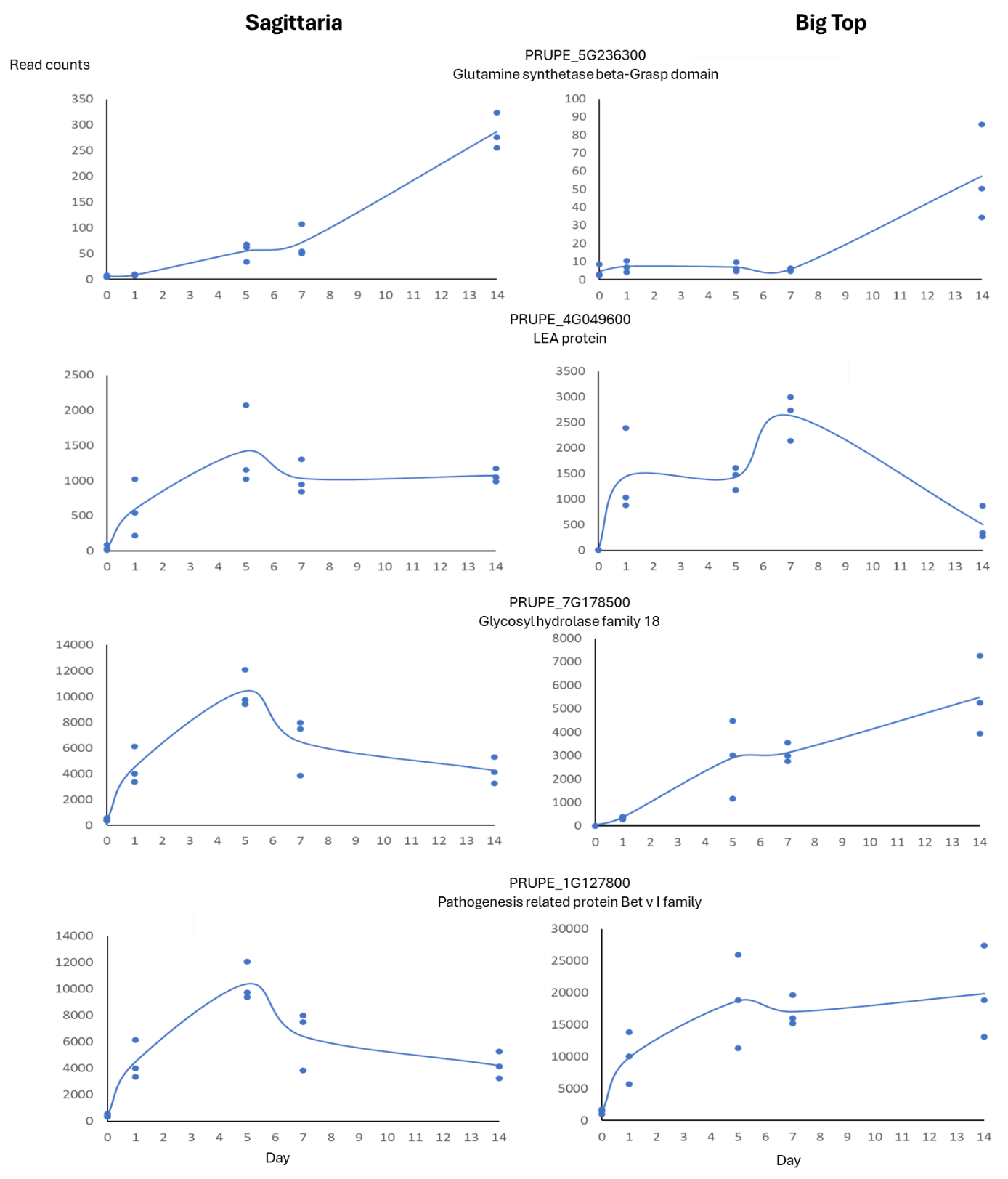


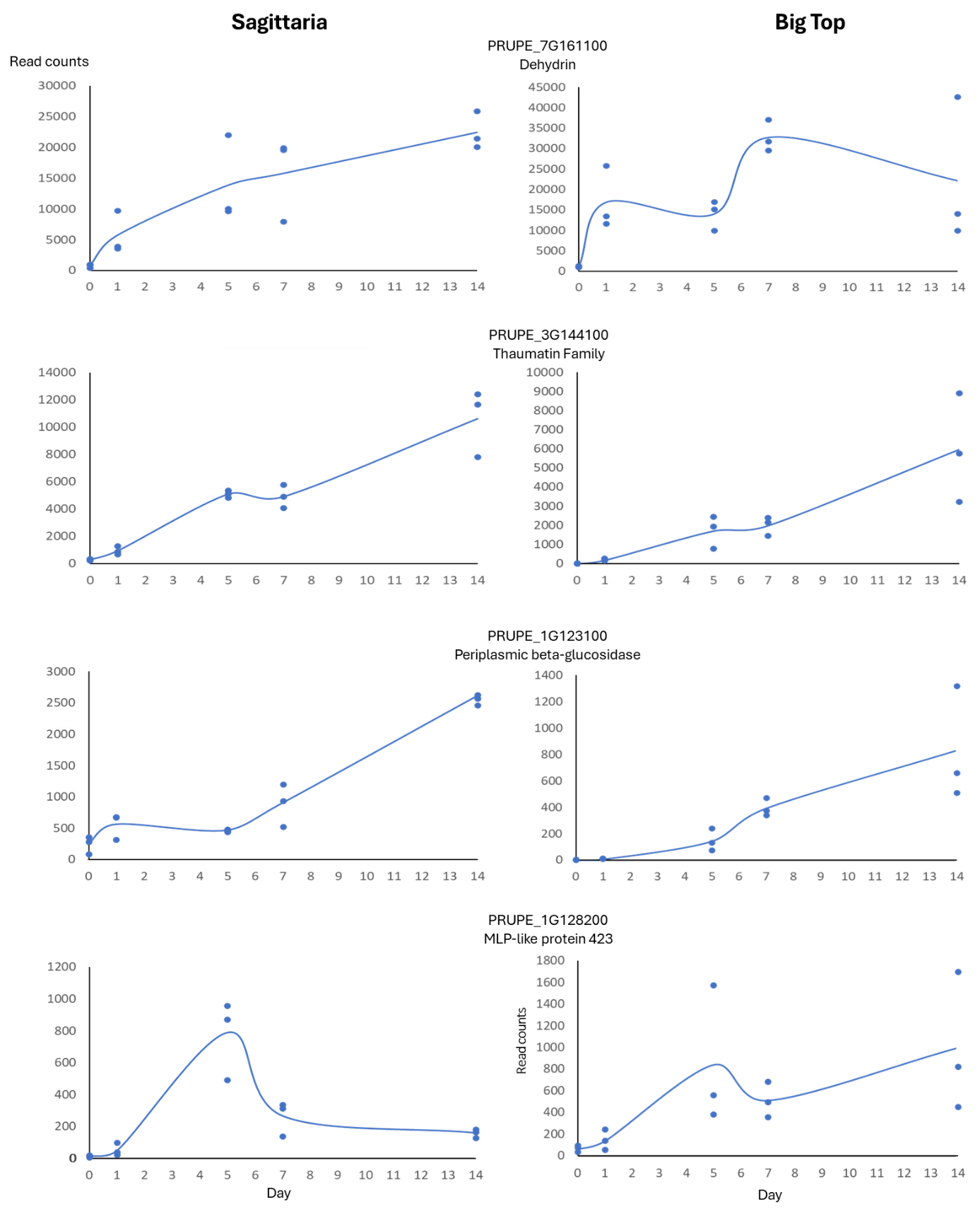


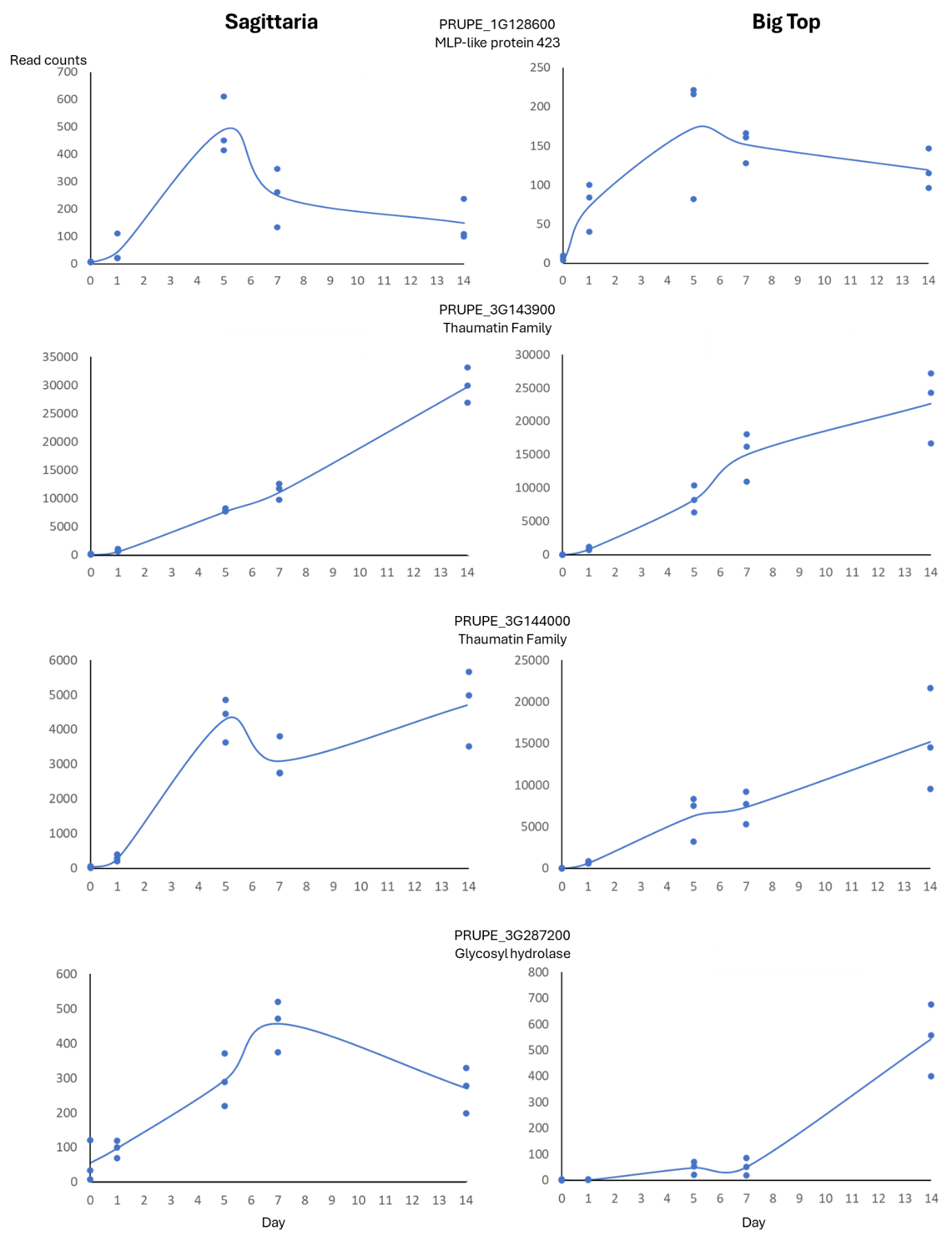


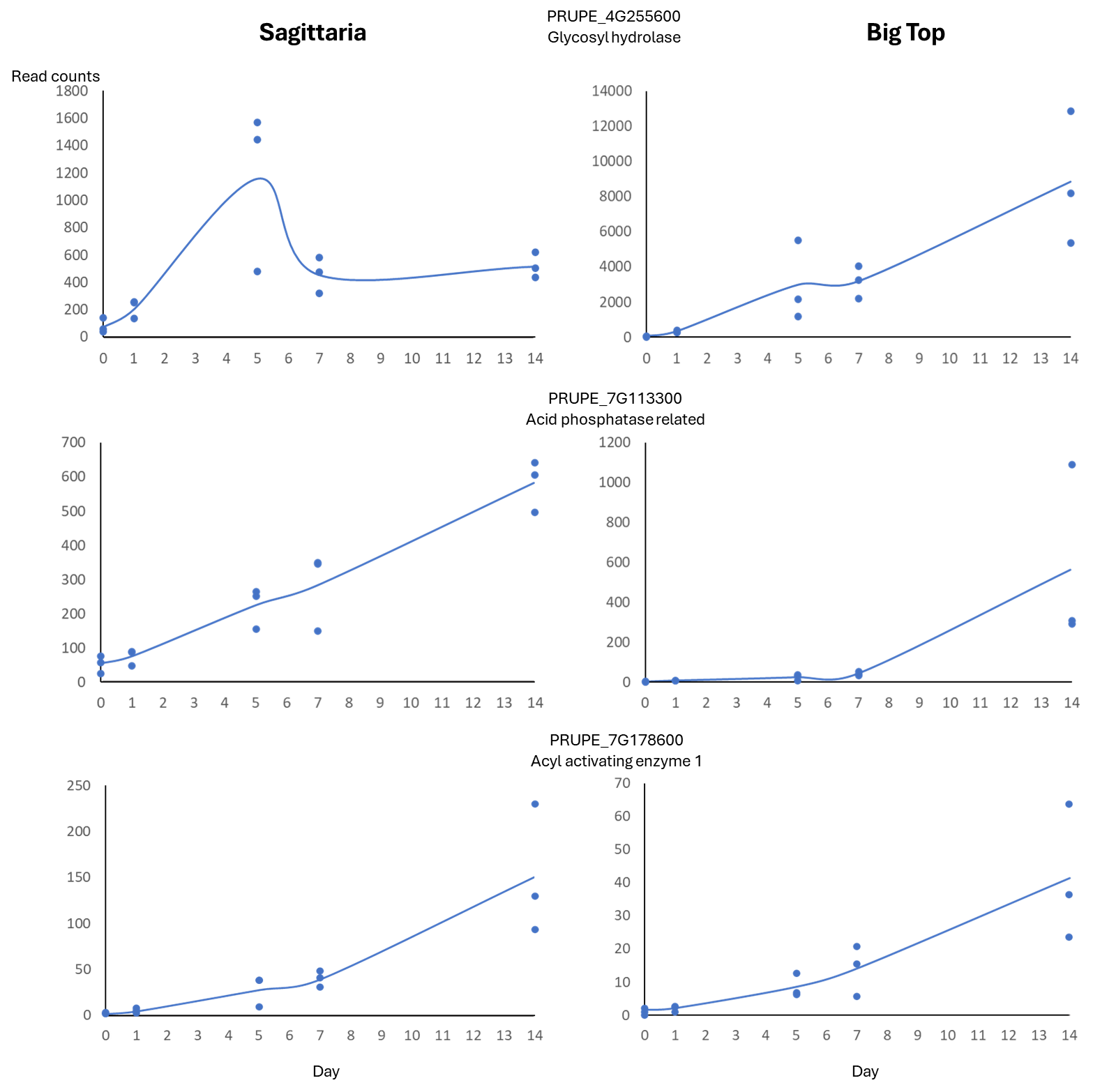


Supplementary Figure S1. Expression pattern in the transcriptome data of 15 potential markers for prolonged chilled storage. Functional annotation based on Muto et al. (2022). Graphs based on the R package ImpulseDE2.

Supplementary Figure S2. ELISA analysis of selected proteins in Sagittaria peach and Big Top nectarine during cold storage treatment at 5 °C at 0, 5, and 14 d followed by 36 h recovery at ambient temperature (22 °C). Concentration in ngmL^-1^: (A) Pathogenesis related protein Bet v I family, (B) Dehydrin, (C) Glycosyl hydrolases family 18, (D) Late Embryogenesis Abundant (plants) LEA-related. Different letters indicate significant differences among cultivars considering all time points. Statistical analyses were performed using Two-way ANOVA and Tukey’s ranked test (P < 0.05). Data are the mean ± SE; n=3.

| **Supplementary Table S1**: Primers used in the present study. Specific primer pairs for selected genes were designed using Primer3 and then Primer-BLAST (Ye et al. 2012) was used to check primer specificity with “*Prunus persica* (taxid:3760)” | | | | |
| --- | --- | --- | --- | --- |
| **GENE ID** | **GENE NAME** | **forward primer (5'-3')** | **reverse primer (5'-3')** | **lenght** |
| Prupe_5G236300 | Glutamine synthetase, beta-Grasp domain | GCTCACACAAACTACAGCACC | GTCTGCTGTTTCATGTCGCC | 159 |
| Prupe_4G049600 | Late Embryogenesis Abundant (plants) LEA-related | GGGACTGTAGTGGATCAGGTCAA | CCAACGCGCCCTCTTTC | 90 |
| Prupe_7G178500 | Glycosyl hydrolases family 18 | CGGGTAATGCAATCGGAAAG | GGCTCCACCTGCCAGTGAT | 149 |
| Prupe_1G127800 | Pathogenesis related protein Bet v I family | AGATGCTCTTTCAGACAAGGTTGA | TCTTTGCCAGCCTTAACATCCT | 149 |
| Prupe_7G161100 | Dehydrin | GCAGGGTACGGCACTCATAC | GTCCTGGAGTTGGCCTAGTAA | 183 |

**Supplementary Table S2:** Transcriptomic data Log2FC for ‘Big Top’ and ‘Sagittaria’ for each of the 15 selected genes

| Big Top | | | | |
| --- | --- | --- | --- | --- |
| Gene ID | Day1/Day0 | Day5/Day1 | Day7/Day5 | Day14/Day7 |
| PRUPE_5G236300 | 0.636 | -0.053 | -0.275 | 3.317 |
| PRUPE_4G049600 | 7.735 | -0.011 | 0.883 | -2.397 |
| PRUPE_7G178500 | 5.656 | 3.078 | 0.108 | 0.819 |
| PRUPE_1G127800 | 2.796 | 0.922 | -0.139 | 0.22 |
| PRUPE_7G161100 | 3.937 | -0.269 | 1.227 | -0.563 |
| PRUPE_3G144100 | 6.203 | 3.218 | 0.218 | 1.58 |
| PRUPE_1G123100 | 2.222 | 3.663 | 1.424 | 1.072 |
| PRUPE_1G128200 | 1.138 | 2.554 | -0.716 | 0.955 |
| PRUPE_1G128600 | 3.397 | 1.209 | -0.194 | -0.344 |
| PRUPE_3G143900 | 6.416 | 3.199 | 0.858 | 0.592 |
| PRUPE_3G144000 | 5.86 | 3.205 | 0.223 | 1.04 |
| PRUPE_3G287200 | 0.102 | 4.998 | 0.079 | 3.399 |
| PRUPE_4G255600 | 3.149 | 3.217 | 0.096 | 1.48 |
| PRUPE_7G113300 | 2.226 | 1.586 | 0.755 | 3.744 |
| PRUPE_7G178600 | 1.002 | 2.003 | 0.716 | 1.553 |
| Sagittaria | | | | |
| PRUPE_5G236300 | 0.585 | 2.779 | 0.381 | 2.009 |
| PRUPE_4G049600 | 3.773 | 1.25 | -0.459 | 0.054 |
| PRUPE_7G178500 | 2.587 | 3.808 | 0.089 | 0.938 |
| PRUPE_1G127800 | 3.326 | 1.209 | -0.693 | -0.606 |
| PRUPE_7G161100 | 2.911 | 1.273 | 0.188 | 0.508 |
| PRUPE_3G144100 | 1.729 | 2.438 | -0.054 | 1.112 |
| PRUPE_1G123100 | 1.204 | -0.268 | 0.942 | 1.53 |
| PRUPE_1G128200 | 1.947 | 3.879 | -1.565 | -0.731 |
| PRUPE_1G128600 | 2.875 | 3.246 | -0.991 | -0.728 |
| PRUPE_3G143900 | 2.156 | 3.252 | 0.52 | 1.399 |
| PRUPE_3G144000 | 2.938 | 3.868 | -0.475 | 0.606 |
| PRUPE_3G287200 | 0.838 | 1.604 | 0.635 | -0.764 |
| PRUPE_4G255600 | 1.42 | 2.448 | -1.342 | 0.18 |
| PRUPE_7G113300 | 0.489 | 1.585 | 0.336 | 1.043 |
| PRUPE_7G178600 | 1.195 | 2.401 | 0.498 | 1.91 |

**Supplementary Table S3:** Transcriptomic data Log2FC between ‘Sagittaria’ and ‘Big Top’ for each day

| Gene ID | Day0 | Day1 | Day5 | Day7 | Day14 |
| --- | --- | --- | --- | --- | --- |
| Prupe_5G236300 | 0.19 | 0.12 | 2.98 | 3.63 | 2.32 |
| Prupe_4G049600 | 2.69 | -1.27 | -0.01 | -1.35 | 1.10 |
| Prupe_7G178500 | 3.96 | 0.90 | 1.63 | 1.61 | 1.73 |
| Prupe_1G127800 | -1.66 | -1.13 | -0.85 | -1.40 | -2.23 |
| Prupe_7G161100 | -0.53 | -1.56 | -0.01 | -1.05 | 0.02 |

**Supplementary Table S4:** Real-time PCR data Log2FC for ‘Big Top’ and ‘Sagittaria’ of selected potential markers during chilled storage at two storage temperatures

| Big Top 1°C | | | | |
| --- | --- | --- | --- | --- |
| Gene ID | Day1/Day0 | Day5/Day1 | Day7/Day5 | Day14/Day7 |
| Prupe_1G127800 | 4.67 | -0.10 | -0.34 | -0.19 |
| Prupe_7G161100 | 6.92 | 0.32 | 1.06 | -3.20 |
| Prupe_7G178500 | 9.82 | 4.18 | -0.20 | -0.34 |
| Prupe_4G049600 | 9.64 | -0.12 | 0.24 | -4.85 |
| Sagittaria 1°C | | | | |
| Prupe_1G127800 | 1.58 | -0.82 | 0.85 | -2.20 |
| Prupe_7G161100 | 4.07 | 1.41 | 1.87 | -1.03 |
| Prupe_7G178500 | 1.72 | 4.00 | 4.52 | -0.68 |
| Prupe_4G049600 | 9.64 | -0.12 | 0.24 | -4.85 |
| Big Top 5°C | | | | |
| Prupe_1G127800 | 3.25 | -0.19 | 2.38 | -3.36 |
| Prupe_7G161100 | 8.28 | -0.54 | 2.34 | -1.70 |
| Prupe_7G178500 | 12.93 | -3.40 | 3.61 | -0.75 |
| Prupe_4G049600 | 9.29 | -0.82 | 3.92 | -4.01 |
| Sagittaria 5°C | | | | |
| Prupe_1G127800 | 5.41 | -0.07 | -0.17 | -0.34 |
| Prupe_7G161100 | 5.70 | 1.77 | -0.15 | -0.99 |
| Prupe_7G178500 | 6.20 | 2.62 | -2.75 | 2.51 |
| Prupe_4G049600 | 11.14 | 0.68 | -1.33 | -0.50 |

**Supplementary Table S5:** Real-time PCR data Log2FC between ‘Sagittaria’ and ‘Big Top’ for each day for the selected genes

| 1 °C | | | | | |
| --- | --- | --- | --- | --- | --- |
| Gene ID | Day0 | Day1 | Day5 | Day7 | Day14 |
| Prupe_1G127800 | 1.96 | -1.14 | -1.86 | -0.67 | -2.69 |
| Prupe_7G161100 | -0.81 | -3.66 | -2.56 | -1.74 | 0.42 |
| Prupe_7G178500 | 5.95 | -2.15 | -2.34 | 2.38 | 2.05 |
| Prupe_4G049600 | 2.67 | -2.77 | -0.29 | 2.30 | 3.42 |
| 5 °C | | | | | |
| Prupe_1G127800 | 1.96 | 4.13 | 4.25 | 1.70 | 4.72 |
| Prupe_7G161100 | -0.81 | -3.38 | -1.06 | -3.56 | -2.85 |
| Prupe_7G178500 | 5.95 | -0.78 | 5.24 | -1.12 | 2.14 |
| Prupe_4G049600 | 2.67 | 4.52 | 6.02 | 0.77 | 4.28 |
